# Approximating the semantic space: word embedding techniques in psychiatric speech analysis

**DOI:** 10.1038/s41537-024-00524-7

**Published:** 2024-12-02

**Authors:** Claudio Palominos, Rui He, Karla Fröhlich, Rieke Roxanne Mülfarth, Svenja Seuffert, Iris E. Sommer, Philipp Homan, Tilo Kircher, Frederike Stein, Wolfram Hinzen

**Affiliations:** 1 Department of Translation & Language Sciences, Universitat Pompeu Fabra, Barcelona, Spain; 2 Department of Psychiatry and Psychotherapy, University of Marburg, Marburg, Germany; 3 Center for Mind, Brain and Behavior (CMBB), Hans-Meerwein-Str. 6, 35032 Marburg, Germany; 4 Department of Neuroscience, University Medical Center Groningen, Antoni Deusinglaan 2, room 117 Groningen, Groningen, Netherlands; 5 Department of Adult Psychiatry and Psychotherapy, University of Zurich, Zurich, Switzerland; 6 Neuroscience Center Zürich, University of Zürich and ETH Zürich, Zürich, Switzerland; 7 Institució Catalana de Recerca i Estudis Avançats (ICREA), Barcelona, Spain

**Keywords:** Biomarkers, Schizophrenia

## Abstract

Large language models provide high-dimensional representations (embeddings) of word meaning, which allow quantifying changes in the geometry of the semantic space in mental disorders. A pattern of a more condensed (‘shrinking’) semantic space marked by an increase in mean semantic similarity between words has been recently documented in psychosis across several languages. We aimed to explore this pattern further in picture descriptions provided by a transdiagnostic German sample of patients with schizophrenia spectrum disorders (SSD) (*n* = 42), major depression (MDD, *n* = 43), and healthy controls (*n* = 44). Compared to controls, both clinical groups showed more restricted dynamic navigational patterns as captured by the time series of semantic distances crossed, while also showing differential patterns in the total distances and trajectories navigated. These findings demonstrate alterations centred on the dynamics of the flow of meaning across the semantic space in SSD and MDD, preserving previous indications towards a shrinking semantic space in both cases.

## Introduction

Semantic relations between words produced during spontaneous speech have been a focal point of interest in recent studies aiming to identify a speech biomarker of psychosis^1–5^ and depression^6^. While this aim can be pursued in a mainly practical perspective (e.g., classification^2,7–9^), it is a theoretical question of considerable interest whether major psychiatric disorders involve a cognitive deviation in the organization of the semantic space. In the case of schizophrenia, this question has been explored since the inception of the neuropsychology of this disorder (for a historical account see refs. ^10,11^). The emergence of large language models (LLM) has provided the opportunity to quantify semantic distances between words through the cosine similarities (angles) between vectorized representations of their meanings (‘embeddings’). These angles reflect human intuitions of how similar two words are in meaning (e.g. with *doctor* more similar to *nurse* than *cat*, say; see refs. ^12,13^); and independently, LLMs have shown marked potential to predict brain activity related to linguistic processing^14–16^. Each vector embedding is a location in a high-dimensional semantic space, and spontaneous speech can be mapped out as a journey between them, whose geometrical properties could illuminate cognitive mechanisms involved.

Since the pioneering proof-of-concept study by Elvevåg and colleagues^17^, a widespread assumption has been that mean semantic similarity metrics derived from LLMs can measure coherence, with the prediction of lower mean similarity in psychosis under the assumption that speech in psychosis is less coherent. Numerous studies have confirmed this prediction^1,3,4,18–21^, though coherence itself has rarely been directly human-rated in the same samples. Aspects of speech quantity may confound semantic similarity metrics as well, as can the choice of different pictures as stimuli for speech elicitation. Recently, multiple findings of *higher* mean semantic similarity (a ‘shrinking’ semantic space) in different cross-sections of SSD and languages have transpired (Turkish:^8,9^; Dutch:^2^; English:^5,22^; Mandarin:^23^; Danish:^4^; see also a verbal fluency result in Mandarin in ref. ^24^ and a result from Russian written texts in ref. ^25^). This new pattern questions the coherence interpretation of mean semantic similarities from LLMs.

He and colleagues^22^ proposed a shift away from the coherence interpretation to using different language models to tap into mechanisms of semantic cognition^26^, exploiting their respective differential internal architectures. Thus, cognitive mechanisms governing lexical-conceptual meaning may be approximated with the token-level model fastText^27^, while meaning at a contextual level as mediated by grammar can be targeted with BERT (Bidirectional Encoder Representations from Transformers^28^) and prompt-response behavior in relation to a visual stimulus can be explored with bimodal models such as CLIP^29^. Using these models comparatively, He and colleagues^22^ found a pattern of increased mean consecutive semantic similarity using non-contextual embeddings from fastText in both first episode psychosis (FEP) and clinical high risk of psychosis (CHR) as compared to controls, which disappeared with contextual models (BERT) in these groups, and with either of these models in a chronic psychosis group capturing a later disease stage. This pattern of a shrinking semantic space using fastText combined with another of increased *unpredictability* of words at the grammatical level (‘perplexity’), and a decreased bimodal semantic similarity (widening of the semantic space) between descriptions and pictures when using CLIP.

Aggregated measures like the mean semantic similarity of consecutive word pairs can capture the tendency of a semantic space to deflate (i.e., shrink) or inflate (expand). But they fail to capture other aspects of the organization of the semantic space such as time-variant dynamic patterns of navigation and global aspects of the geometry of the space. As for the dynamic patterns, these can be approximated by plotting the semantic distances crossed between consecutive embeddings as the points of a time-series (from the distance between points 1 and 2 to those between points 2 and 3, 3 and 4, etc.). From these ‘waves’, formal properties can be extracted such as the number of times that the average line of semantic similarities is crossed (mean crossing rate), the rate of slope sign changes (semantic similarities increasing after decreasing, or vice versa), and the autocorrelation (predictability from itself) of this series. A shrinking semantic space could be reflected in a higher autocorrelation, less crossing, and less slope sign changes of this series, without this being necessarily reflected in changes in mean semantic similarity.

Regarding the global geometry of the semantic space, this can be captured through the centroids of all the embeddings generated from a transcript (their weighted averaged vector, identifying the central semantic point of the space), as well as through the embeddings’ dispersion around these centroids (i.e., how spread-out they are around this central point: dispersion is high if embeddings are scattered far and wide). Recently, Xu et al.^30^, computed the semantic distances of each semantic unit’s vector to the centroids both statically and dynamically (i.e., as semantic information is accumulated in the centroid unit by unit). Results suggested AUCs > 0.70 when predicting human ratings of derailment for centroid-based measures only, exceeding those based on mean or minimal semantic similarities. Beyond centroids and dispersion, the total displacement involved in a trajectory through the high-dimensional semantic spaces mapped by LLMs can be measured as the sum of the Euclidean distances between embeddings crossed. The overall geometric shapes of the trajectories can be represented through the total volumes and areas of the polyhedrons formed by all the embeddings jointly. Note that it is possible to obtain the same values in the same aggregated measures (e.g., mean semantic similarity) in two speech samples or groups, even if the trajectories and global organization of the spaces are different. For example, if a trajectory has low dispersion and high average semantic similarity, the volume will be small, while with the same high average semantic similarity but large dispersion, it will have a larger volume. Volume and dispersion thus provide complementary insights, suggesting that frequently used measures of mean semantic similarity may be unstable surface manifestations of a deeper underlying distortion of the semantic space.

The primary aim of this study was to expand beyond static / aggregated measures commonly used so far to dynamic and global geometric measures capturing trajectories through space, controlling for both speech quantity and picture effects. We pursued this in a transdiagnostic German group of patients comprising both schizophrenia spectrum disorders (SDD) and major depressive disorder (MDD), where computational semantic studies have been largely absent so far (but see ref. ^6^). Our hypothesis was that in the SDD sample, even if evidence for a shrunk semantic space as measured through mean semantic similarity would not be seen (as in the chronic sample of ref. ^22^), such evidence might transpire in dynamic and global measures. Our study of MDD was exploratory.

## Results

Results are reported from a sample of 129 German-speaking individuals: patients diagnosed with schizophrenia and schizoaffective disorder (SSD, *n* = 42), major depressive disorder (MDD, *n* = 43), and healthy controls (HC, *n* = 44)^31^, which were age, sex and IQ-matched (Table 1). Speech samples were obtained through the task of describing four images of the Thematic Apperception Test (TAT)^32^. Word embeddings for each speech sample were obtained utilizing fastText and BERT. At the level of sentences, we employed the SentenceTranformers framework^33^, specifically using the ‘aari1995/German_Semantic_STS_V2’ model, which provides a 1024-dimensional vector for each sentence.

**Table 1:** Demographics of participants and comparisons.

	HC	MDD	SSD	MDD - HC	MDD - SSD	HC - SSD
	(mean ± SD)	(mean ± SD)	(mean ± SD)	(*p*-value)	(*p*-value)	(*p*-value)
N° participants	44	43	42			
Age (years)	42.9 ± 12.3	42.1 ± 12.5	40.5 ± 11.9	0.7860	0.5491	0.3766
Female (%)	32%	37%	38%	0.7616	1.0000	0.7008
Education (years)	14.7 ± 3.3	12.7 ± 2.6	11.9 ± 2.0	0.0028**	0.0922	0.0000***
IQ	117.5 ± 14.2	114.6 ± 13.8	111.6 ± 15.9	0.3502	0.3563	0.0786
Age of onset (years)	-	25.1 ± 12.9	17.7 ± 8.2	-	-	-

IQ scores are derived from the MWT-B scale, which assesses verbal premorbid intelligence. Gender comparisons were performed using the χ² test, while *t*-tests were used for comparing other variables. Significance levels are denoted as follows: *p < 0.05, **p < 0.01, ***p < 0.0001.

### Preliminary analyses

**Fig. 1:**
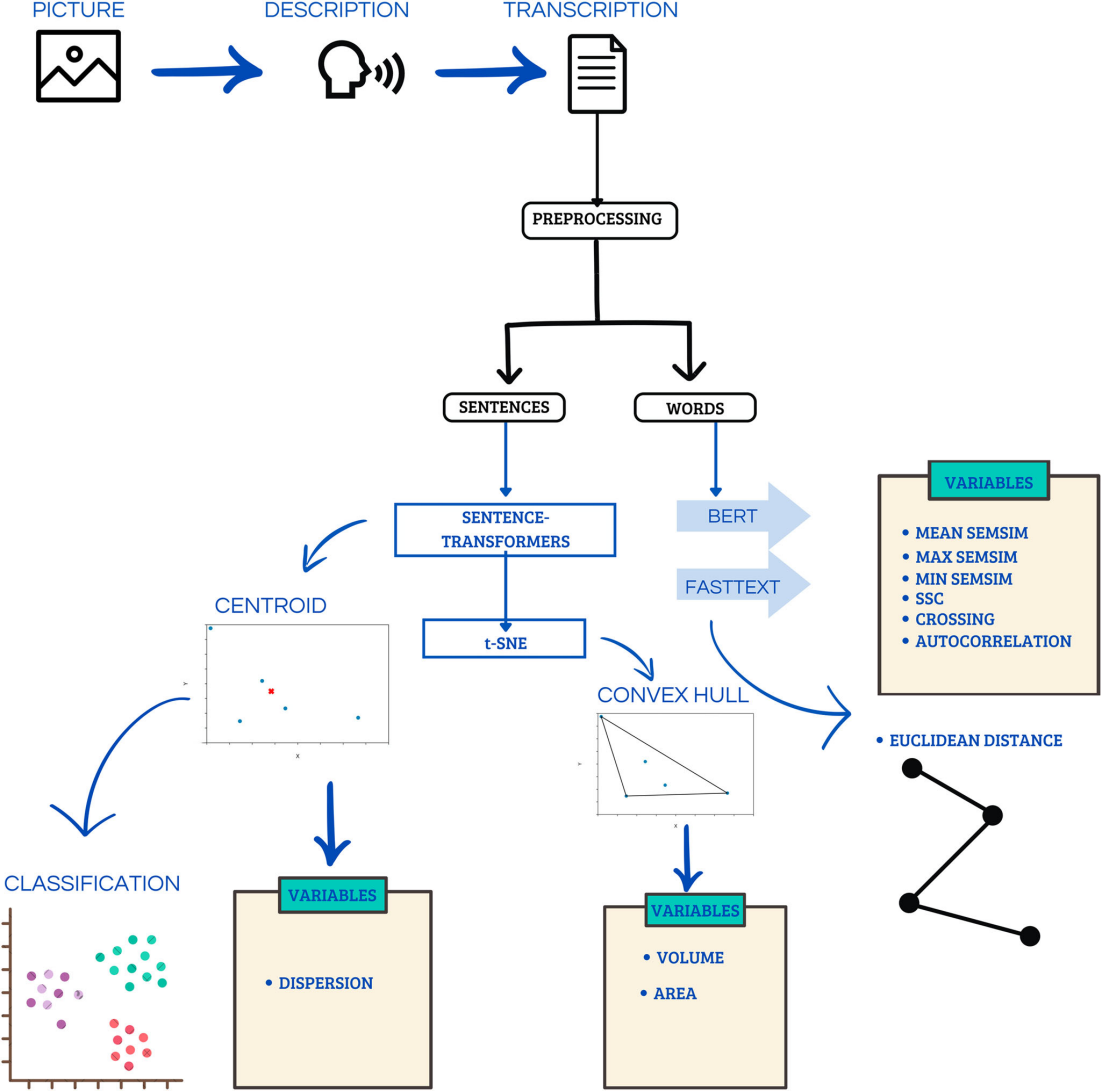
Analytical pipeline. Participants describe pictures, and their speech is transcribed and preprocessed. Sentences and words are analyzed separately. Sentence embeddings are generated using Sentence-Transformers and visualized using t-SNE to compute centroids and dispersion. Word embeddings are extracted using BERT and FastText to construct convex hulls and calculate their volume and area. Mean semantic similarity, maximum and minimum similarity, slope sign changes (SSC), crossings, and autocorrelation, are derived from embeddings. Euclidean distances between points in the semantic space are also calculated. These variables are used for classification and further analysis.

**Fig. 2:**
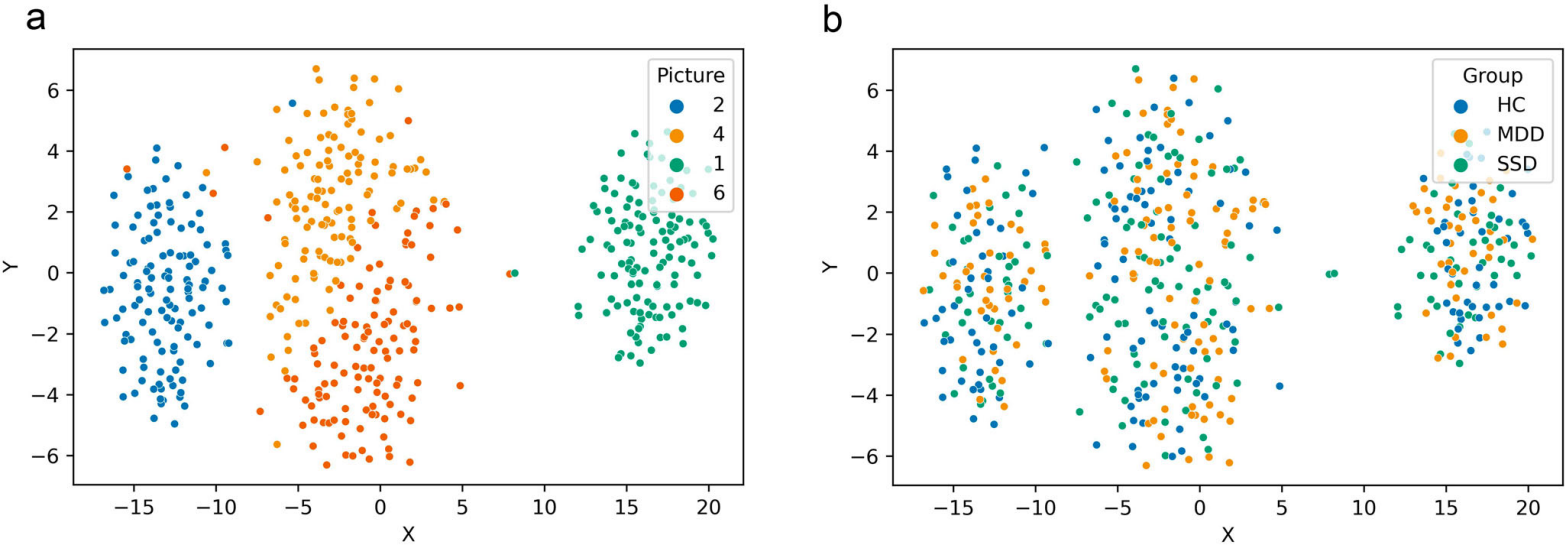
Classification of text centroids from sentence embeddings in 2D. Sentence embeddings for each picture description were reduced to two dimensions using t-SNE, with centroids visualized for each speech. For the same speech centroids, in panel (**a**), colors indicate each picture description, and in panel (**b**), colors indicate group.

Our analytical pipeline is schematically presented in Fig. 1. In a preliminary and baseline semantic analysis, we calculated sentence embedding centroids for each text sample and investigated length effects on semantic similarity metrics. Centroids were calculated as in ref. ^30^, based on the average values for each dimension. After reducing 1024-dimensional embeddings into a 2-dimensional space with t-Distributed Stochastic Neighbor Embedding (t-SNE)^34^, a k-nearest neighbors (kNN) algorithm was used to classify pictures and groups. This algorithm showed near-ceiling performance for pictures, but chance performance for groups (Fig. 2): Accuracy=96.6%, precision (picture 1: 100%, picture 2: 98.4%, picture 4: 92.2%, picture 6: 95.7%), recall (picture 1: 99.2%, picture 2: 99.2%, picture 4: 96.7%, picture 6: 91.1%), average specificity=98.9%, and f1 score (picture 1: 99.6%, picture 2: 98.8%, picture 4: 94.4%, picture 6: 93.3%). Metrics for the performance of group classification were: Accuracy=64.9%, precision (MDD: 62.5%, HC: 64.3%, SSD: 70.6%), recall (MDD: 71.9%, HC: 77.6%, SSD: 44.7%), average specificity=82.4%, and f1 score (MDD: 66.9%, HC: 70.3%, SSD: 54.8%). These results indicate that while speech centroids based on sentence embeddings successfully distinguished between different pictures, thus capturing their inherent semantics, groups did not differ in terms of this baseline semantics. Put differently, when describing the same picture, different speech samples are rooted in the same semantic space and comparable for this reason. Figure 2 visualizes the centroids of each speech sample as reduced into a two-dimensional space.

**Fig. 3:**
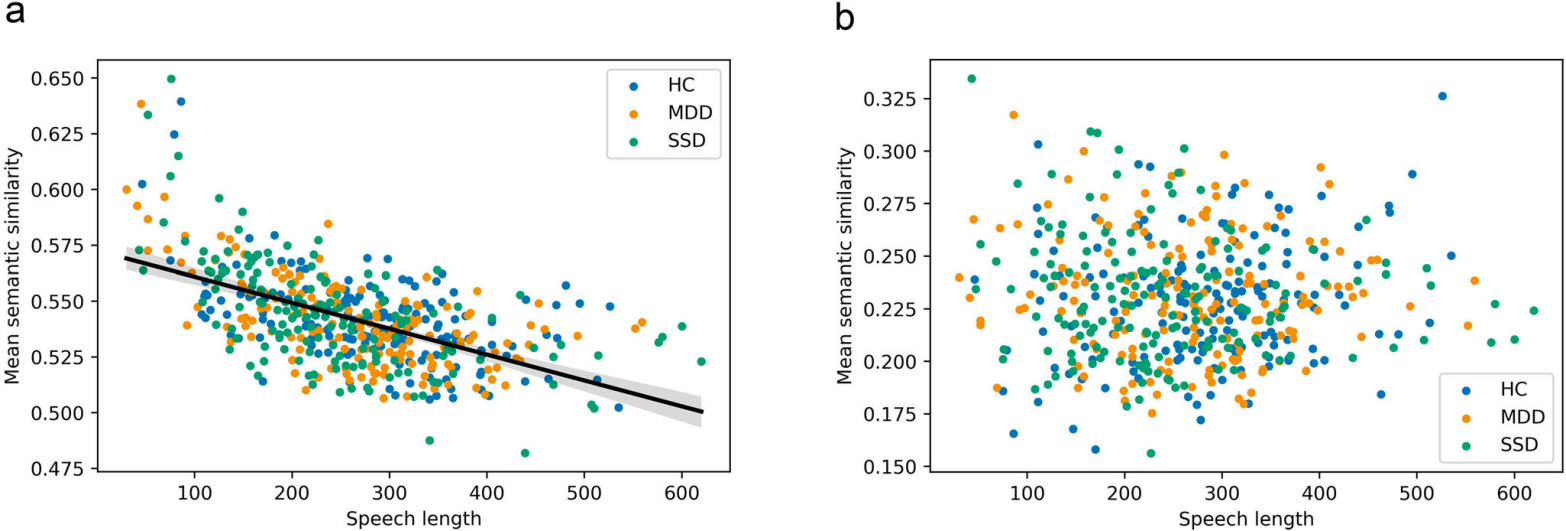
Mean semantic similarity versus word count for all pictures and participants. In panel (**a**), using BERT model a negative correlation is depicted. In panel (**b**), no correlation was observed using fastText model.

As a second preliminary step, we determined possible effects of speech quantity (word count and average sentence length) on semantic similarity assessments. Table S1 shows the total number of words for each participant and picture as well as average sentence lengths. In all pictures, mean length was higher in HC compared to both MDD and SDD (and higher in the MDD group compared to the SSD group). However, using t-tests Bonferroni-corrected for multiple comparisons to determine groups effects, only differences in mean sentence length in Pictures 2 and 4 (HC > SSD) remained statistically significant (corrected *p*-value: 0.0391 and 0.0278, respectively; see Figure S1). In addition, a negative correlation was observed between word count and mean semantic similarity when utilizing BERT (Pearson correlation, r = −0.57, *p* < 0.001), but not when using fastText (Pearson correlation, *r* = −0.001, *p* = 0.977). The same negative correlation was observed within each group when using BERT, and only in SSD when using fastText (Pearson correlation, *r* = −0.16, *p* < 0.05) (see Fig. S2). Figure 3 presents the correlation in a scatter graph for all groups with each point representing a single speech sample. Unlike with word count, there was no significant correlation between mean semantic similarity and average sentence length across all groups (using BERT, Pearson correlation, *r* = −0.05, *p* = 0.238; using fastText, Pearson correlation, *r* = −0.02, *p* = 0.511). These differences and their interaction with semantic similarity suggest speech quantity to be a possible confounding variable, which needs to be controlled when comparing groups for semantic similarity metrics.

### Static and dynamic semantic analysis

**Fig. 4:**
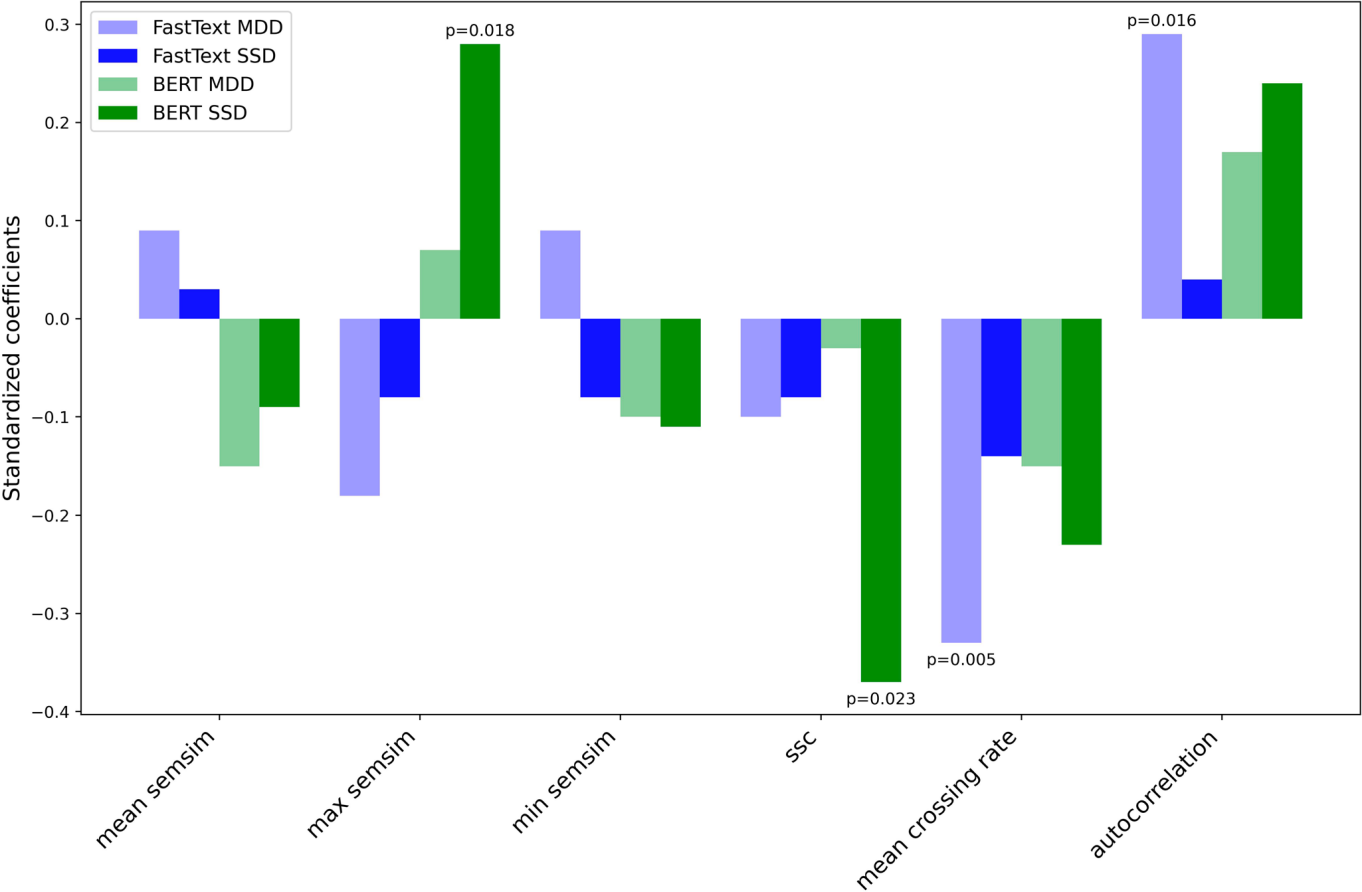
Summary of group effects on semantic similarity variables. The plot displays standardized coefficients for the group variables, indicating group effects from separate regressions for each variable. Only significant coefficients are shown, with their corresponding p-values displayed next to the bars. Blue bars represent results for fastText, with light blue indicating MDD and blue indicating SSD. Green bars represent results for BERT, with light green for MDD and green for SSD.

Figure 4 summarizes group effects for semantic similarity variables, showing the standardized coefficients for the group variable and the *p*-values that were significant at the 0.05 level (additionally, an initial exploratory analysis comparing semantic similarity variables using a Kruskal-Wallis test is shown in Table S2). These results are derived from mixed-effects regression models for each dependent variable, which included both word count and average sentence length as predictors (see Eq. (1), *Methods*). In all regressions, Picture 1 and HC were used as baselines. Table S3 contains all the standardized coefficients and *p*-values, while detailed summaries for each regression can be found in Table S4. There were no significant group differences in mean semantic similarity, but there was a significant increase in maximum semantic similarity in SSD with respect to HC when using the BERT model. The remaining results clearly show a distinct pattern for MDD and SDD: in particular, significant differences were observed for MDD only when using fastText, while in SSD this was the case only for BERT. Specifically, MDD showed a lower mean crossing rate and higher autocorrelation, while SSD showed less sign slope changes (SSC). A trend in SSD was observed for a lower mean crossing rate (*p* = 0.087) and higher autocorrelation (*p* = 0.084) as well. Together, these dynamic metrics indicate that the time series of semantic similarities is more stable in the clinical groups. Additionally, the analysis revealed that the individual pictures had a significant effect on the semantic variables, as shown in Table S4. This highlights that in addition to speech quantity, picture effects need to be taken into account when computing semantic similarity related metrics, as was done here in all of our mixed effect models.

### Displacement and dispersion of semantic trajectories

We defined total displacement as the total length of a trajectory starting from a given location, measured as the cumulative Euclidean distances between word embeddings using the 300 dimensions of the fastText model. Table 2 shows larger displacement in SSD relative to HC, despite the unchanged centroids and mean semantic similarities as reported above (see Eq. (1), *Methods*). Unlike with semantic similarities, displacement exhibited no picture effect. The number of content words and average sentence length co-predicted displacement. To further explore the relationship between these predictors and the displacement variable, we ran the same regression with mean displacement (cumulative Euclidean distance divided by the number of content words) as the dependent variable. The results were also significant and in the same direction (see Table S5). There was no statistically significant difference in displacement across groups at the sentence level (see Table S6).

**Table 2:** Summary of mixed linear model regression results for total displacement.

	Coefficient	Std. Error	z	*p* > |z|	95% CI
Intercept	−16.469	4.813	−3.422	0.001	[−25.902, 7.036]
MDD	7.363	4.463	1.650	0.099	[−1.384, 16.109]
SSD	18.649	4.516	4.516	0.000	[9.798, 27.501]
Picture 2	−1.099	1.793	-0.613	0.540	[−4.613, 2.415]
Picture 4	1.980	1.672	1.184	0.236	[−1.297, 5.258]
Picture 6	0.409	1.701	0.240	0.810	[−2.925, 3.743]
Content words	1.199	0.036	33.655	0.000	[1.129, 1.268]
Av sentence length	−0.407	0.130	-3.139	0.002	[−0.661,−0.153]

The coefficients presented are unstandardized. Degrees of freedom = 485.

Next, the dispersion of a semantic space measures how spread-out sentence embeddings are relative to the centroid (average point). As seen in Table 3, MDD showed higher dispersion compared to HC in sentence embeddings with respect to the centroids of the speech samples (see Eq. (2), *Methods*). That is, although the centroids of the sentence embeddings thematically identified the different pictures and did not distinguish groups, as noted, sentences in MDD were more dispersed with respect to these centroids. Despite controlling for the number of sentences in the regression models, a negative effect of length was seen, with a greater number of sentences associating with lower dispersion.

**Table 3:** Summary of mixed linear model regression results for dispersion.

	Coefficient	Std. Error	z	*p* > |z|	95% CI
Intercept	0.707	0.010	68.545	0.000	[0.687, 0.728]
MDD	0.015	0.005	2.817	0.005	[0.005, 0.026]
SSD	0.008	0.005	1.536	0.124	[-0.002, 0.019]
Picture 2	−0.022	0.006	−3.703	0.000	[−0.033, −0.010]
Picture 4	0.013	0.006	2.147	0.032	[0.001, 0.024]
Picture 6	−0.003	0.006	−0.465	0.642	[−0.014, 0.009]
Sentences	−0.003	0.000	−9.423	0.000	[−0.003,−0.002]
Av sentence length	−0.001	0.000	−1.782	0.075	[−0.001, −0.000]

The coefficients presented are unstandardized. Degrees of freedom = 485.

### Convex hulls

Finally, the convex hull of a given space of embeddings is a boundary capturing the extent and the outer limits of the distribution of embeddings, showing the furthest reaches of the sentence meanings in the semantic space. The boundary encloses all the embeddings in the tightest possible way, like a rubber band wrapped around them. With each sentence embedding serving as a vertex derived from the convex hull of all points, we calculated areas and hyper-volume (hereafter, ‘volume’) of the hyper-polyhedrons resulting, while varying the number of dimensions to gauge the sensitivity of this parameter (see Table S8 for this sensitivity analysis). Dimensionality reduction was necessary due to the requirement of having at least (*n* + 1) points (in this case, sentence embeddings) to construct a convex hull in *n* dimensions. Therefore, we mapped the original 1024 dimensions for each sentence embedding into a space of 5 dimensions, accounting for the number of sentences in each speech sample (mean = 19, SD = 8.2).

Our mixed-effect model (Eq. (3), *Methods*) considered the random effect of participants and the fixed effects of the group and the number of sentences produced. It revealed a significant decrease in the volume of the convex hull in SSD compared to HC (Table 4). When the analysis was repeated with the area of the convex hull as the dependent variable, SSD exhibited a smaller area as well, consistent with the volume decrease (*p* = 0.027) (see Table S7). Instead of regressing the volume directly, we first calculated the natural logarithm of the volume and then regressed this variable. This transformation stabilized the variance and normalized the distribution, allowing us to interpret the coefficients as percentage changes (see further *Methods*).

**Table 4:** Summary of mixed linear model regression results for the natural logarithm of volume in a 5-dimensional space.

	Coefficient	Std. Error	z	*p* > |z|	95% CI
Intercept	−1.905	0.759	−2.508	0.012	[−3.393, −0.416]
MDD	−0.256	0.643	−0.398	0.690	[−1.517, 1.005]
SSD	−1.426	0.647	−2.205	0.027	[−2.693, −0.158]
Picture 2	0.717	0.513	1.397	0.162	[−0.289, 1.723]
Picture 4	0.722	0.512	1.412	0.158	[−0.280, 1.725]
Picture 6	0.810	0.509	1.591	0.112	[−0.188, 1.808]
Sentences	0.487	0.031	15.935	0.000	[0.427, 0.547]

The coefficients presented are unstandardized. Degrees of freedom = 468.

Table S8 shows the results of a sensitivity analysis using various numbers of dimensions (ranging from 3 to 9) to which the original 1024 dimensions were reduced. While the results remain largely consistent, it is important to note that increasing the number of dimensions will inevitably exclude an increasing number of speech samples with fewer sentences. Conversely, including speech samples with fewer sentences necessitates reducing the number of dimensions significantly, which leads to a loss of information.

### Correlations to clinical scores

**Fig. 5:**
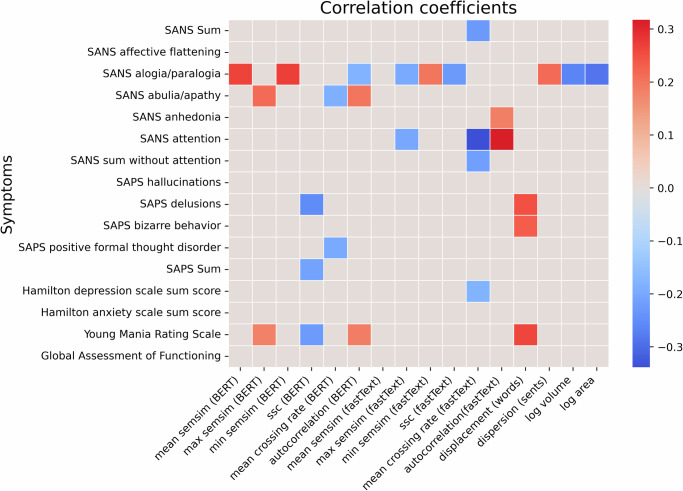
Significant correlation coefficients between variables and clinical symptoms. The heatmap illustrates the correlations between analyzed variables and clinical measures, including SAPS and SANS symptoms, the Hamilton Depression Scale (sum score), the Hamilton Anxiety Scale (sum score), the Young Mania Rating Scale, and the Global Assessment of Functioning. Colors range from blue (negative correlations) to red (positive correlations), with only significant coefficients displayed.

Significant correlations between the variables under analysis, which were averaged across pictures, and various symptoms scale scores are displayed in Fig. 5. Analogous correlational analyses for each picture separately can be found in Figure S3.

## Discussion

We set out to enhance our understanding of the structure and geometry of the semantic space as spanned by the words produced in the course of a picture description task, with particular reference to a pattern of a shrinking semantic space in psychosis as previously documented in a number of languages [2, 5, 8, 9. 22]. In this latter respect, results showed that clinical groups did not significantly differ from controls in either embedding centroids or mean semantic similarity. However, there was higher maximum semantic similarity in SSD, consistent with a shrinking semantic space. Lack of sensitivity in mean semantic similarity replicates the finding of^22^ in their chronic SSD sample, and raises the question to what extent higher semantic similarity is an effect in early psychosis only (first episode and clinical high risk, as in ref. ^22^). In addition to disease stage, failure to control for picture effects and speech quantity may be a factor contributing to a contradictory picture in semantic similarity metrics. In our study, the picture chosen for speech elicitation as well as word count made a difference to semantic similarity measurements, though a word count effect was only seen for the BERT model; within-groups, it was only seen in SSD, and it was weak. Crucially, even when including picture, word count and sentence length in the model, results show that dispersion, displacement, and the convex hull volume and area retain significance between groups. This may indicate that these variables are more robust across these confounds.

In addition to word count and sentence length, more evidence for task effects is required. Unlike free speech, a picture description task restricts the semantic content of spontaneous speech to the content of the picture seen, rather than letting the mind roam more freely, as could be the case in a free speech task where topics and topic transitions are less restricted. Pictures may thus inadvertently serve as an external restriction to the very phenomenon we intend to observe. In line with this, recent studies that found higher semantic similarity in SSD^2,8,9^ featured spontaneous speech as elicited by questions in semi-structured interviews. On the other hand^22^, used picture descriptions, and a number of previous studies have reported decreased semantic similarity in free speech samples, using earlier generations of language models^17–19^. Task comparisons are a critical desideratum if carrying NLP-based classification is to be carried over to clinical applications.

We confirmed our prediction that the navigational dynamics would distinguish groups even when mean semantic similarities or centroids do not. Clinical groups showed differences in the time series of consecutive semantic distances crossed: MDD showed higher autocorrelation and less crossing of the average line than controls, while SSD showed less slope sign changes. This evidence is again consistent, now at a dynamic level of analysis, with a shrinking semantic space in SSD and extending this theme to MDD: in both groups, a more stable or restricted pattern is seen, in which it becomes more predictable how semantic distances are crossed in sequence and how larger distances are crossed after shorter ones or vice versa. Neurotypical language production might involve universal patterns with respect to this predictability and variability, which deserves closer scrutiny in its own right, along with a disruption of these patterns in major mental disorders.

Cosine similarities are higher-order metrics which collapse the high dimensionality of semantic embedding spaces into a single value. By contrast, Euclidean distances preserve the dimensionality and geometrical relationships between points. Relative to controls, SSD not only showed a distinct dynamic pattern in crossing semantic distances as measured with cosine similarities between consecutive words, but travelled greater distances in total, as measured as the sum of Euclidean distances as well (higher displacement). This suggests more erratic behavior at the word level, which is not picked up in mean cosine semantic similarity. In addition, at the level of sentences, SDD showed smaller volumes and areas of their convex hulls, demonstrating a difference in the global geometry of the semantic spaces they build, despite not differing in their central points (centroids). This again reinforces, at this third analytical level, the finding of the shrinking semantic space (more condensed volumes and areas).

Together, these results reinforce the methodological message that aggregated measures such as centroids or means of semantic similarity may fail to capture changes in the geometry of the semantic spaces that exist at a dynamic and global geometric level. These latter analytical dimensions identify different patterns of thought and meaning construction in language, which may provide crucial input for a neurocognitive model of mental disorders, long after the term ‘loosening of association’ was coined and investigated through associative lexical relations between words^10^. While this concept has never been extended to MDD so far, overlaps in the dynamic patterns between MDD and SDD – and specific patterns such as greater dispersion in MDD relative to controls – suggest rich future potential insights from cross-diagnostic studies for changes in semantic cognition as manifest in their linguistic readouts in spontaneous speech.

While automatic semantic analysis has been suggested to pick up subthreshold clinical symptoms that are not picked up in clinical ratings of symptom-dimensions^1,35^, our correlational results did reveal significant (though weak) associations between dynamic measures (total displacement) and positive symptoms in particular (SAPS delusions and bizarre behavior). Intriguingly, these correlations were positive, suggesting higher displacement with more erratic thought (delusions) and behavior. Nonetheless, a pattern of significant associations with negative symptom dimensions (SANS alogia) was seen in more than half of our measures, with both negative associations in the case of dynamic measures and global geometry (mean crossing rate, autocorrelation and SSC of the time series, volume and area of the convex hulls), and positive associations in the case of mean, minimum and maximum semantic similarity. Based on this it is intriguing to speculate that the more alogia is clinically detected, the more the semantic space contracts, as per higher mean semantic similarity and a narrower band of minimum and maximum semantic similarity, as well as lesser SSC and lesser volumes and areas of the convex hulls; while at the same time, the time series is more predictable (higher autocorrelation). It is the conjunction of our measures that therefore contributes to the contraction, seeing and reinforcing it from different perspectives. As cautionary note against this speculation, the correlational analysis does not take into account confounding factors such as speech quantity, which we have seen affect our semantic variables.

Although this sample did not include additional measures of cognitive functioning, such measures would allow us to explore their relationship with various semantic variables. For instance, they could help determine whether static and dynamic variables correspond to distinct cognitive constructs. However, we cannot yet anticipate different predictions for static and dynamic variables, as both are designed to capture the behavior of the ‘wave’ function characterizing semantic similarity between consecutive words. Further exploration is needed, including correlations with predictability measures such as surprisal and next-word prediction.

A final aspect of our results is that semantic variables based on BERT embeddings were only sensitive in the case of SSD, while those based on fastText were only sensitive in MDD. This confirms the need to approximate the semantic space using different language models that, due their internal architectures, are sensitive to different aspects of linguistic organization and semantic cognition^22^. BERT embeddings are contextual, capturing not only individual meaning of words but also their structural relationships. Ignoring structure, in other words, and restricting ourselves to the lexical-conceptual semantic level, reveals changes in MDD but not SSD, where the disruption may be more prominent at a structural level – at least in this type of task. Free conversational speech might extend lexical-conceptual deficits to SDD.

## Conclusions

This study found that, while both clinical groups were rooted in the same semantic space as shown through centroids and did not show differences in aggregated measures such as mean semantic similarities, they diverged in dynamic patterns of crossing semantic distances over time and in the global geometries of their spaces. These findings reinforce patterns of a shrinking semantic space for both disorders, while opening a new hypothesis space for the mechanistic investigation of how meaning is mapped through language, when our thought capacity undergoes major neuropathological changes.

## Methods

### Data collection

The dataset comprised recordings from 129 German-speaking individuals: patients diagnosed with schizophrenia and schizoaffective disorder (SSD, *n* = 42), major depressive disorder (MDD, *n* = 43), and healthy controls (HC, *n* = 44)^31^. Demographic and clinical characteristics of the sample are summarized in Table 1, along with differences between groups. Table 1 shows that the only significant demographic difference is in years of education, with the HC differing from both the SDD and MDD groups. The Scale for the Assessment of Negative Symptoms (SANS)^36^ and the Scale for the Assessment of Positive Symptoms (SAPS)^37^ were used to examine clinical factors related to negative and positive symptoms, respectively.

Speech samples were obtained through the task of describing four images of the Thematic Apperception Test (TAT)^32^ using the methodology outlined for assessing the Thought and Language Index (TLI)^38^. The speech samples consisted of descriptions to the four depicted images (SM). The expected speech duration for describing each picture set for each participant was three minutes. Transcriptions were conducted manually to ensure a verbatim representation. For the present study, annotations of pauses, prolonged silences, or hesitations were additionally removed, resulting in a mean of 264.2 words per picture and participant (SD = 102.2). The TAT test provided a controlled environment for analysing speech, as each picture guides the participants to describe specific topics, limiting lexical and thematic variability. Table S1 contains the descriptions of the mean speech length and mean sentence length for each group and picture. Additionally, significant differences between groups at each picture level are presented in the SM.

Ethical permissions for the data collection and sharing of the data for the present study were obtained from the Ethik-Kommission des Fachbereichs Humanmedizin der Philipps-Universität Marburg (AZ 07:14).

### Extracting word and sentence embeddings

First, word embeddings for each speech sample were obtained utilizing fastText^27^ and BERT^28^. When calculating linguistic variables derived from fastText embeddings, we removed stopwords and punctuations. This process was carried out using spaCy (version 3.6.0) and the ‘de_core_news_lg’ model for German. As for BERT embeddings, we included all words and utilized a pre-trained model for German (‘dbmdz/bert-base-german-cased’), which was fine-tuned for each sample.

At the level of sentences, we employed the SentenceTranformers framework^33^ to calculate embeddings for each sentence in every picture description. Specifically, we used the ‘aari1995/German_Semantic_STS_V2’ model, which provides a 1024-dimensional vector for each sentence^39^. demonstrated that this framework outperforms averaged word-level embeddings for unsupervised sentence similarity evaluation.

### Variables derived from word and sentence embeddings

For a complete list of all semantic variables calculated, see Table 5. We calculated sentence embedding centroids for each text sample, as previously proposed by ref. ^30^. These centroids were calculated as the average value for each dimension. This served to explore whether centroids could effectively distinguish either groups or pictures, as well as to ensure comparability of semantic content as such between the groups, against which group differences in dynamic navigational patterns could then be profiled. We reasoned that centroids can act as representative embeddings of speech samples, capturing their semantic essence or topic. Additionally, for each sample, we measured the dispersion of sentence embeddings with respect to the centroid.

**Table 5:** List and descriptions of variables.

Variable	Description
mean semsim	Average of consecutive semantic similarity scores
max semsim	Maximum of consecutive semantic similarity scores
min semsim	Minimum of consecutive semantic similarity scores
ssc	Count of slope sign changes in the wave function divided by the number of scores
mean crossing rate	Frequency at which the wave function intersects its mean value
autocorrelation	First-order autocorrelation in the wave function
displacement (words)	Cumulative Euclidean distance between word embeddings
displacement (sentences)	Cumulative Euclidean distance between sentence embeddings
dispersion	Dispersion of sentence embeddings around a speech sample’s centroid
volume	hypervolume enclosed by the sentence embeddings’ convex hull
area	Surface area of the sentence embeddings’ convex hull

Semantic similarity was assessed within text samples at the word level. For fastText word embeddings, this focused solely on content words, while for BERT embeddings, all words were included. For each consecutive pair of either words or sentences, the mean, maximum, and minimum semantic similarity values within each sample were computed (‘static’ semantic variables). Furthermore, we constructed a wave function representing the time series of the semantic similarity values between successive pairs of items. From this function, we derived ‘dynamic’ semantic variables: slope sign change (SSC), autocorrelation, and the frequency at which the wave function intersects its mean value (mean crossing rate). Both ‘static’ and ‘dynamic’ semantic offer distinct ways to characterize the wave function. While static variables, such as the mean, maximum, and minimum, provide aggregated statistical measures that describe the distribution of consecutive similarity values without considering their temporal order, dynamic variables account for the evolution of these values over time. Although this set of variables is not exhaustive, they all aim to capture the same underlying phenomenon. Through these measures, we seek to approximate the behavior of consecutive similarity values.

Taking a step beyond the wave function, we utilized the raw embeddings in the fastText model and computed their total displacement as cumulative Euclidean distances between consecutive word vectors. An analogous procedure was employed at the sentence level. By choosing to directly utilize the embeddings, our aim was to minimize potential information loss involved in aggregating variables such as mean or variance.

Finally, we represented each speech sample by constructing a hyper-polyhedron with each sentence embedding serving as a vertex derived from the convex hull of all points. To facilitate analysis, we employed *t*-Distributed Stochastic Neighbor Embedding (t-SNE) for dimensionality reduction^34^. This allowed us to calculate the convex hull for each sample and subsequently determine the hypervolume of each hull. Dimensionality reduction was necessary due to the requirement of having at least (*n* + 1) points (in this case, sentence embeddings) to construct a convex hull in *n* dimensions. Therefore, we mapped the original 1024 dimensions for each sentence embedding into different spaces, considering the number of sentences in each speech sample (mean = 19, SD = 8.2). We varied the number of dimensions to assess the sensitivity of this parameter (ranging from 3 to 9 dimensions) (see Table S8), and present results here for 5 dimensions. Furthermore, we utilized this technique for visualization purposes to show the centroids of each sample (see Fig. 3).

### Statistical analysis

After obtaining centroids of sentence embeddings for each speech sample and reducing dimensions using t-SNE, a k-nearest neighbors (kNN) algorithm was used to classify pictures and groups. Additionally, we calculated conventional metrics derived from the confusion matrix to assess the performance of this classification (accuracy, precision, recall, average specificity, and f1 score).

Given the complexity of the data, it was important to account for the various factors that could influence the results. To address this, we used mixed-effects models to handle repeated observations within individuals. This allowed us to not only control for participant-level variability but also ensure that group-level differences were estimated more accurately^40^. Specifically, to determine group differences between semantic variables, we ran mixed-effects model to estimate population-averaged effects controlling for picture, speech sample length and average sentence length. Specifically, we predicted the individual variables as a function of group (${MDD}$ and ${SSD}$ as dummy variables), word count (${words}$), average sentence length (${sent\_length}$) and picture described (${{picture}}_{j}$ as dummy variable for pictures 2, 4 and 6, leaving picture 1 as reference), where ${\mu }_{i}$ measured the random effects associated with each participant (see Eqs. (1), (2), and (3)). In the equations the variable *y* represents the different individual semantic variables calculated. Unlike Eq. (1), Eq. (2) contains sentence count (${sentences}$) instead of word count. We used Eq. (3) to determine differences in volume and area of the convex hull. Instead of regressing these variables directly, we regressed their natural logarithm, normalizing the distribution, and leading to more reliable and interpretable results. Additionally, by transforming the variables, the coefficients can be interpreted as approximate percentage changes.

1 $$\begin{array}{l}{y}_{{ij}}={\beta }_{0}+{\beta }_{{MDD}}\times {{MDD}}_{{ij}}+{\beta }_{{SSD}}\times {{SSD}}_{{ij}}+{\beta }_{2}\times {{picture}}_{2}+{\beta }_{4}\times {{picture}}_{4}\\\qquad+\,{\beta }_{6}\times {{picture}}_{6}+{\beta }_{{words}}\times {{words}}_{{ij}}+{\beta }_{{sent}{{\_}}{length}}\times {{sent}{{\_}}{length}}_{{ij}}{+{\mu }_{i}+\varepsilon }_{{ij}}\end{array}$$

2 $$\begin{array}{l}{y}_{{ij}}={\beta }_{0}+{\beta }_{{MDD}}\times {{MDD}}_{{ij}}+{\beta }_{{SSD}}\times {{SSD}}_{{ij}}+{\beta }_{2}\times {{picture}}_{2}+{\beta }_{4}\times {{picture}}_{4}\\\qquad+\,{\beta }_{6}\times {{picture}}_{6}+{\beta }_{{sentences}}\times {{sentences}}_{{ij}}+{\beta }_{{sent}{{\_}}{length}}\times {{sent}{{\_}}{length}}_{{ij}}{+{\mu }_{i}+\varepsilon }_{{ij}}\end{array}$$

3 $$\begin{array}{l}{\mathrm{ln}}{y}_{{ij}}={\beta }_{0}+{\beta }_{{MDD}}\times {{MDD}}_{{ij}}+{\beta }_{{SSD}}\times {{SSD}}_{{ij}}+{\beta }_{2}\times {{picture}}_{2}+{\beta }_{4}\times {{picture}}_{4}\\\qquad+\,{\beta }_{6}\times {{picture}}_{6}+{\beta }_{{sentences}}\times {{sentences}}_{{ij}}\,{+{\mu }_{i}+\varepsilon }_{{ij}}\end{array}$$

As shown in the previous equations, in the analysis of sentence displacement and dispersion, the total number of sentences instead of word count was used. Finally, we ran correlations between each variable and the SAPS and SANS symptoms scales, as well as the Hamilton Depression scale, Hamilton Anxiety scale, Young Mania Rating scale, and Global Assessment of Functioning scale. All the calculations and coding were performed in Python (version 3.10.9).

## Supplementary information

Supplementary material

Supplementary Table S4

Supplementary Table S8

## Data Availability

All data used for the present analyses are available. Please contact FS for access.

## Code availability

The code used to support these findings is available upon request from the corresponding author.
