# Supplementary material for: Approximating the semantic space: word embedding techniques in psychiatric speech analysis
Source: Schizophrenia (Heidelb). 2024 Dec 2;10(1):114. doi: 10.1038/s41537-024-00524-7 (PMC11612388; doi:10.1038/s41537-024-00524-7)
Supplement: Supplementary file 1 — Supplementary material [file 41537_2024_524_MOESM1_ESM.pdf]

Supplementary materials

Pictures from TAT used as prompt

The pictures used as prompt for the speech sample were taken from the Thematic Apperception Test (Murray, 1943), specifically Picture 1, Picture 2, Picture 4BF, and Picture 6. Participants were specifically instructed to create stories based on what they saw in each picture, including details about the character’s thoughts, feelings, and motives.

Length of speech and semantic similarity measures

In a preliminary analysis, we calculated the speech length by counting the number of words for each participant and picture. This information is presented in Table S1, alongside a summary of the average sentence length for each case.

Table S1. Mean speech length (± SD) and mean sentence length (± SD).

Picture	Group	Mean speech length ± SD	Mean sentence length ± SD
1	HC	270.9 ± 102.7	15.9 ± 6.4
	MDD	242.2 ± 105	14.8 ± 6.7
	SSD	220.8 ± 120.3	14 ± 6.8
2	HC	292.9 ± 90	18.2 ± 10.8
	MDD	281.5 ± 95.8	15.2 ± 5.9
	SSD	262.5 ± 103.1	13.5 ± 4.5
4	HC	280.8 ± 102.2	16 ± 6.9
	MDD	267 ± 100.1	14.9 ± 7
	SSD	242.9 ± 112.4	12.6 ± 4.4
6	HC	284.8 ± 97.5	17.6 ± 12.5
	MDD	271.9 ± 101.2	16.3 ± 8.5
	SSD	249.8 ± 107.5	14.3 ± 6.5

We used a t-test to determine whether there were significant differences between groups in both mean length and mean sentence length for each picture. Subsequently, we corrected for multiple comparisons using the Bonferroni method. In all pictures, the mean length of speech was consistently higher in HC group compared to both the MDD and SDD groups (and higher in the MDD group compared to the SSD group). These differences were statistically significant in picture 1 for speech length (HC > SSD) (p-value 0.0443), and in pictures 2 and 4 for average sentence length (HC > SSD) (p-value 0.0130, and p-value

0.0093, respectively). After Bonferroni corrections, only differences in mean sentence length remained significant (picture 2: corrected p-value 0.0391; picture 4: corrected p-value 0.0278). These comparisons are visualized in Figure S1, where significant differences using corrected p-values lower than 0.05 are represented by '*', and non significant differences are represented by 'ns'.

Figure S1. Speech length and mean sentence length differences between groups, for each picture. (A): Picture 1, (B): Picture 2, (C): Picture 4, (D): Picture 6.

A

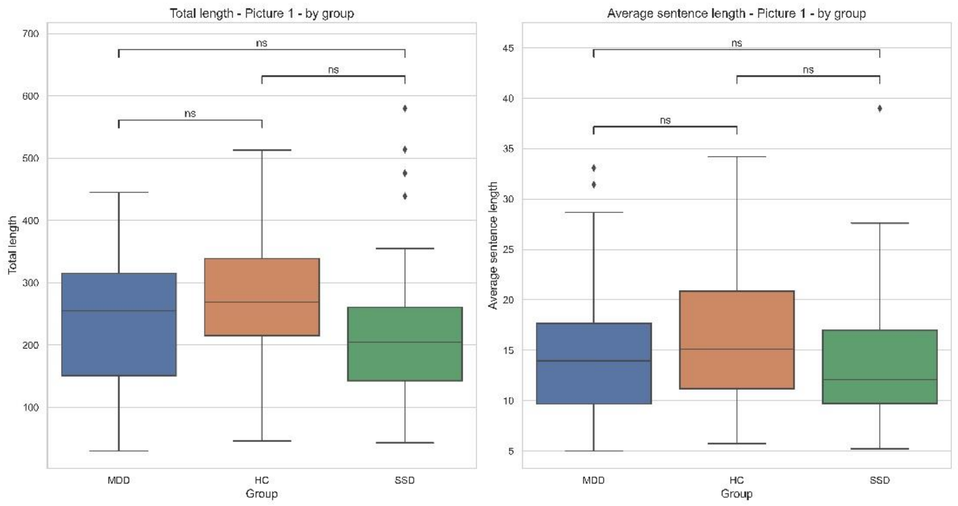

B

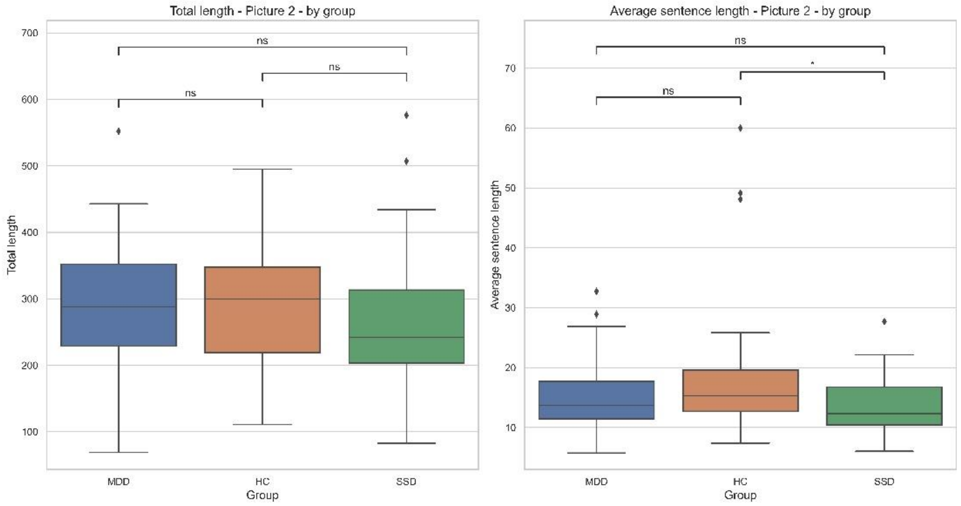

C

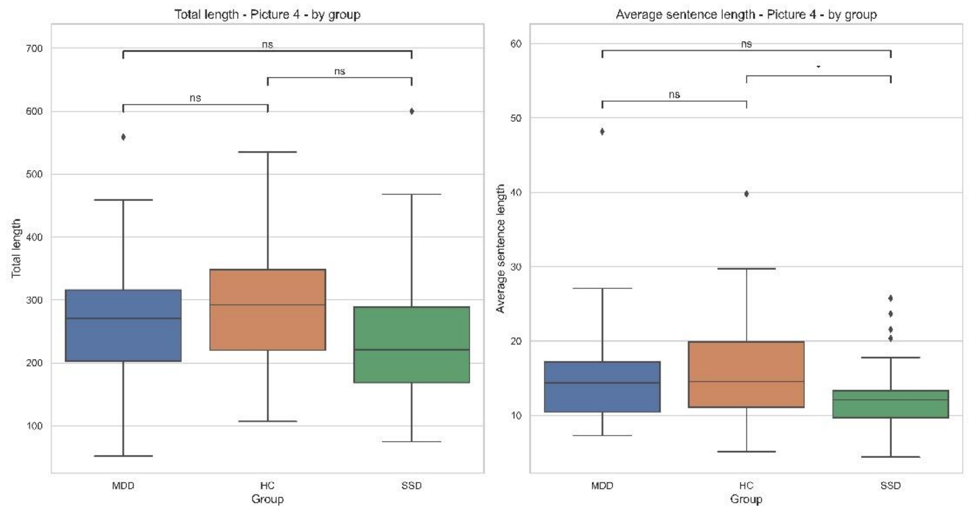

D

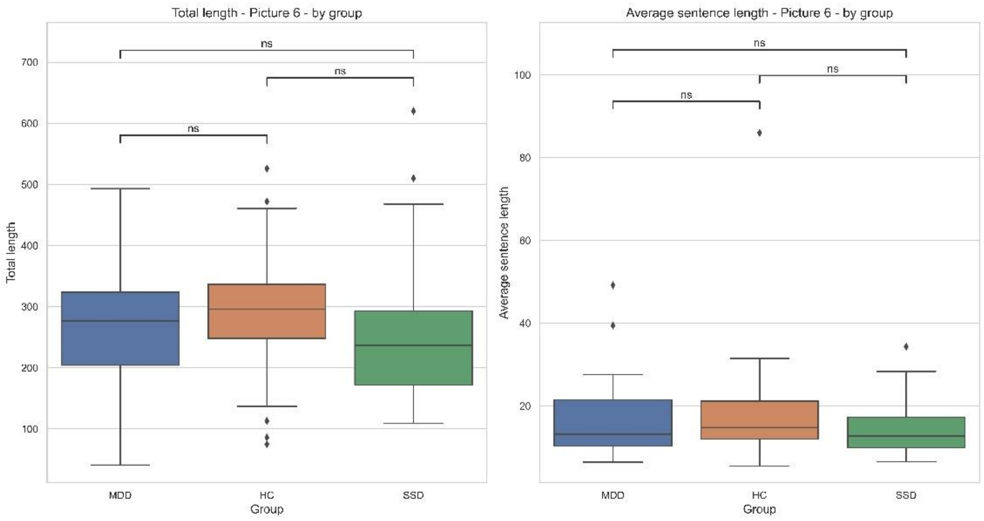

Figure S2 shows scatter graphs of mean semantic similarity versus speech length, for each group and model, where each point represents a single speech sample. A significant negative correlation was observed in each group when using BERT (Pearson correlation, HC: r = -0.52, p < 0.001, MDD: r = -0.59, p < 0.001, SSD: r = -0.58, p < 0.001), and also in SSD, but weak, when using fastText (Pearson correlation, r = -0.16, p = 0.042). MDD also showed a weak negative correlation using fastText (Pearson correlation, r = -0.13, p = 0.093). Correlation was next to null in HC using fastText (Pearson correlation, r = 0.06, p = 0.474).

Figure S2. Mean semantic similarity versus speech sample for all pictures, by model and group.

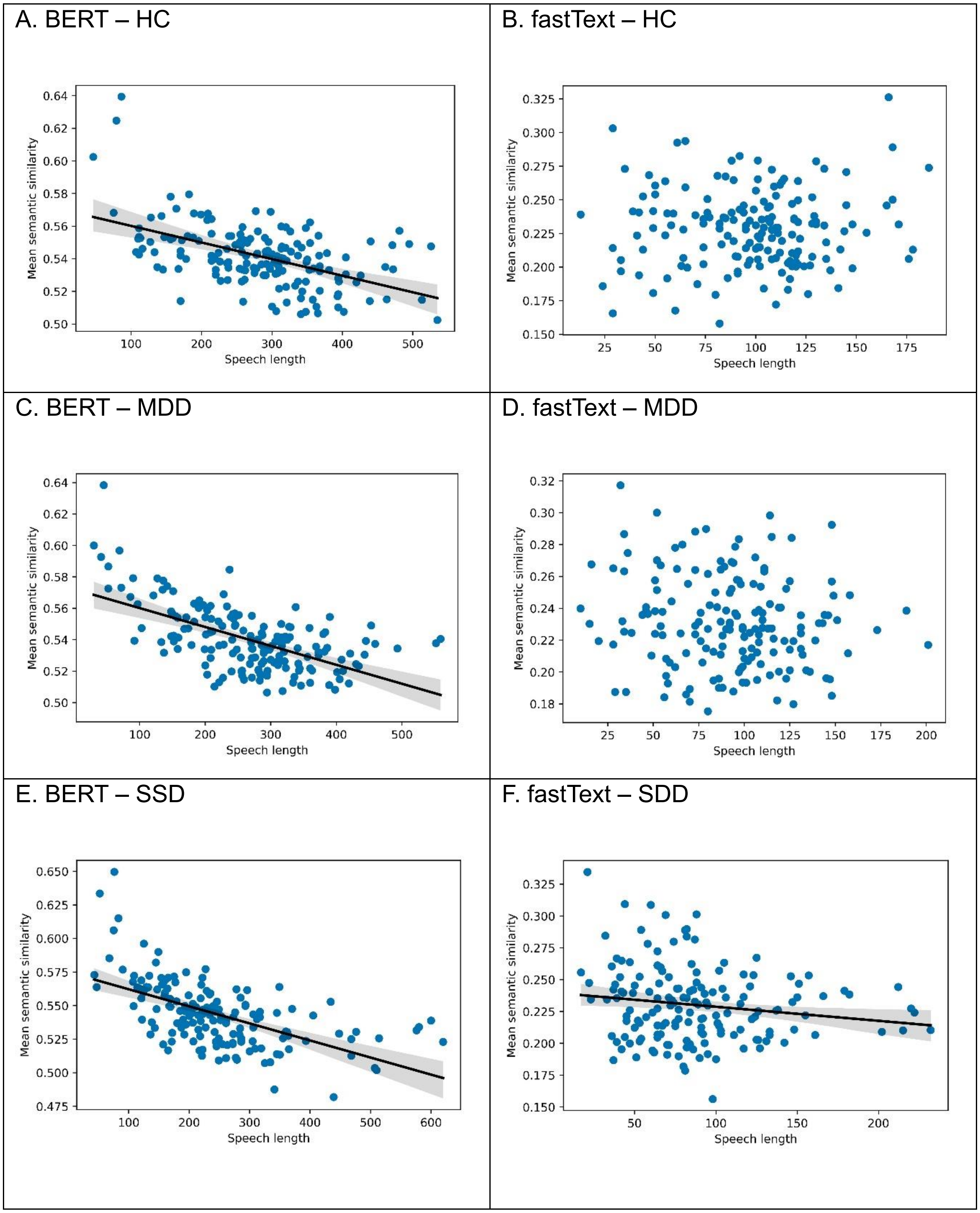

The findings presented align with those reported in Çabuk et al. (2024). In their study with Turkish speakers, the authors showed that individuals with schizophrenia produced shorter sentences compared to control subjects. Additionally, using word2vec embeddings, they found higher semantic similarity in schizophrenia.

Kruskal-Wallis test for semantic similarity measures

Table S2 summarizes the results obtained using the Kruskal-Wallis test, which evaluated group differences in semantic similarity variables derived from the different word embeddings. The results highlight significant differences in maximum semantic similarity (FastText), average SSC (BERT), average crossing (FastText), and autocorrelation (FastText).

Table S2. Kruskal-Wallis test results for semantic variables.

	**FastText**		**BERT**	
**Variable**	**H statistic**	**p-value**	**H statistic**	**p-value**
**mean semsim**	0.483	0.786	3.163	0.206
**max semsim**	6.421	0.040	1.174	0.556
**min semsim**	1.610	0.447	0.414	0.813
**ssc**	4.078	0.130	8.752	0.013
**mean crossing rate**	11.003	0.004	1.653	0.438
**autocorrelation**	8.390	0.015	1.016	0.602

Table S3. Summary of groups effects on semantic similarity variables for each regression y in equation (1).

	**FastText**				**BERT**			
	**MDD**		**SSD**		**MDD**		**SSD**	
**Variable**	**std coeff**	**p-value**	**std coeff**	**p-value**	**std coeff**	**p-value**	**std coeff**	**p-value**
**mean semsim**	0.09	0.541	0.03	0.835	-0.15	0.258	-0.09	0.520
**max semsim**	-0.18	0.105	-0.08	0.478	0.07	0.549	0.28	0.018

**min semsim**	0.09	0.392	-0.08	0.464	-0.10	0.383	-0.11	0.336
**ssc**	-0.10	0.417	-0.08	0.493	-0.03	0.856	-0.37	0.023
**mean crossing rate**	-0.33	0.005	-0.14	0.241	-0.15	0.240	-0.23	0.087
**autocorrelation**	0.29	0.016	0.04	0.733	0.17	0.224	0.24	0.084

Table S4. Regression results for each semantic variable as variable y in Equation (1).
Note: The coefficients presented are unstandardized

Table S5. Summary of mixed lineal model regression for average Euclidean distances between word embeddings.

	Coefficient	Std. Error	z	*p* > |z|	95% CI
**Intercept**	0.875	0.048	18.370	0.000	[0.782, 0.969]
**MDD**	0.081	0.043	1.905	0.057	[-0.002, 0.164]
**SSD**	0.172	0.043	3.987	0.000	[0.087, 0.256]
**Picture 2**	0.012	0.019	0.616	0.538	[-0.025, 0.049]
**Picture 4**	0.036	0.018	2.017	0.044	[0.001, 0.070]
**Picture 6**	0.026	0.018	1.427	0.153	[-0.010, 0.061]
**Content words**	0.001	0.000	3.580	0.000	[0.001, 0.002]
**Av sentence length**	-0.004	0.001	-3.104	0.002	[-0.007, -0.002]

Note: The coefficients presented are unstandardized. Degrees of freedom = 485.

Table S6. Summary of mixed lineal model regression for Cumulative Euclidean distances between sentence embeddings.

	Coefficient	Std. Error	z	*p* > |z|	95% CI
**Intercept**	-0,522	0,751	-0,696	0,487	[-1.994, 0,949]
**MDD**	-0.794	0.480	-1.657	0.098	[-1.734, 0.145]
**SSD**	-0.418	0.487	-0.859	0.390	[-1.373, 0.536]
**Picture 2**	1.522	0.306	4.976	0.000	[0.922, 2.121]
**Picture 4**	-1.345	0.303	-4.440	0.000	[-1.939, -0.751]
**Picture 6**	-0.544	0.304	-1.789	0.074	[-1.139, 0.052]
**Sentences**	2.592	0.023	114.702	0.000	[2.548, 2.636]
**Av sentence length**	-0.033	0.022	-1.478	0.139	[-0.077, 0.011]

Note: The coefficients presented are unstandardized. Degrees of freedom = 485.

Table S7. Summary of Mixed Linear Model Regression results for area of convex hull.

	Coefficient	Std. Error	z	*p* > |z|	95% CI
**Intercept**	5.319	0.391	13.600	0.000	[4.533, 6.086]
**MDD**	-0.225	0.317	-0.711	0.477	[-0.846, 0,395]
**SSD**	-0.703	0.318	-2.211	0.027	[-1.327, -0.080]
**Picture 2**	0.624	0.286	2.180	0.029	[0.063, 1.184]
**Picture 4**	0.544	0.285	1.909	0.056	[-0.015, 1.103]
**Picture 6**	0.619	0.284	2.180	0.029	[0.062, 1.175]
**Sentences**	0.236	0.016	14.984	0.000	[0.205, 0.267]

Note: The coefficients presented are unstandardized. Degrees of freedom = 485.

Table S8. Regression results for ln(volume) with various dimensionality reductions of 1024-D sentence embeddings.

Note: The coefficients presented are unstandardized.

Figure S3. Significant correlation coefficients between variables and clinical symptoms, for each picture.

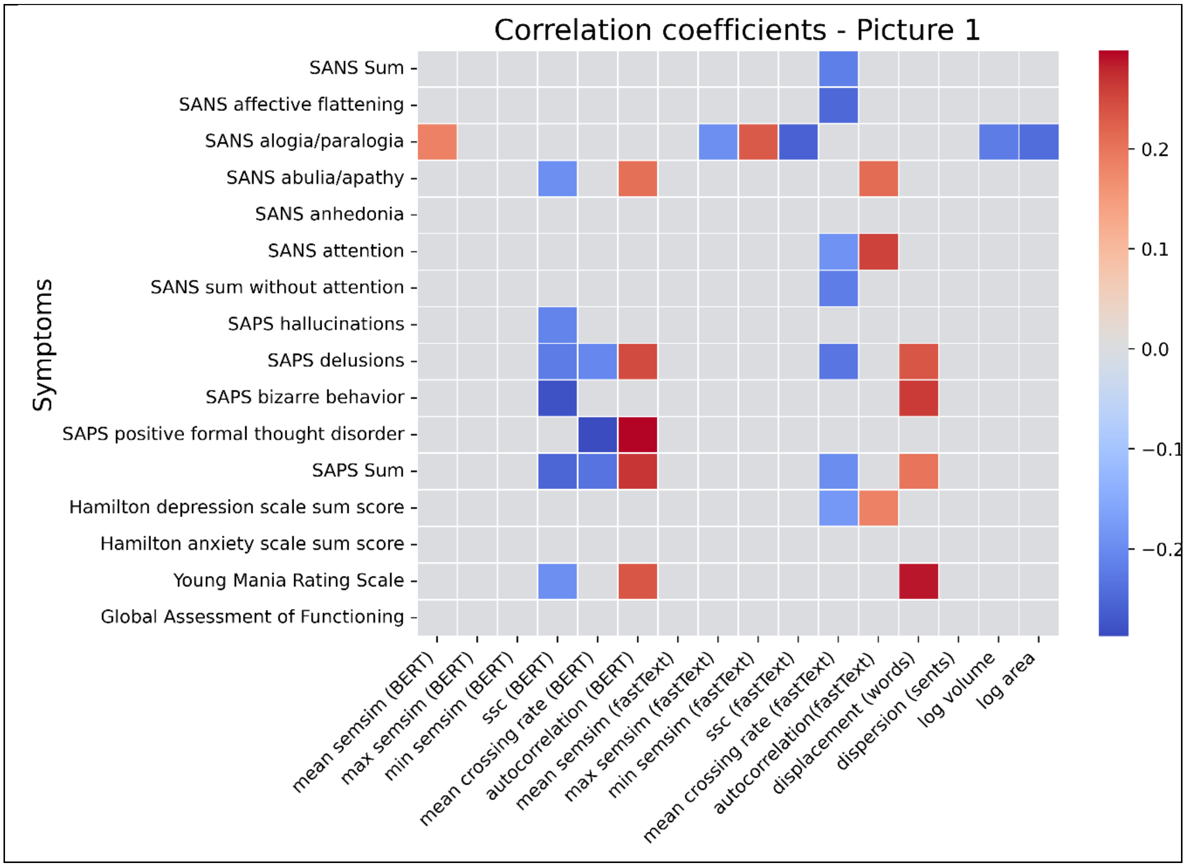

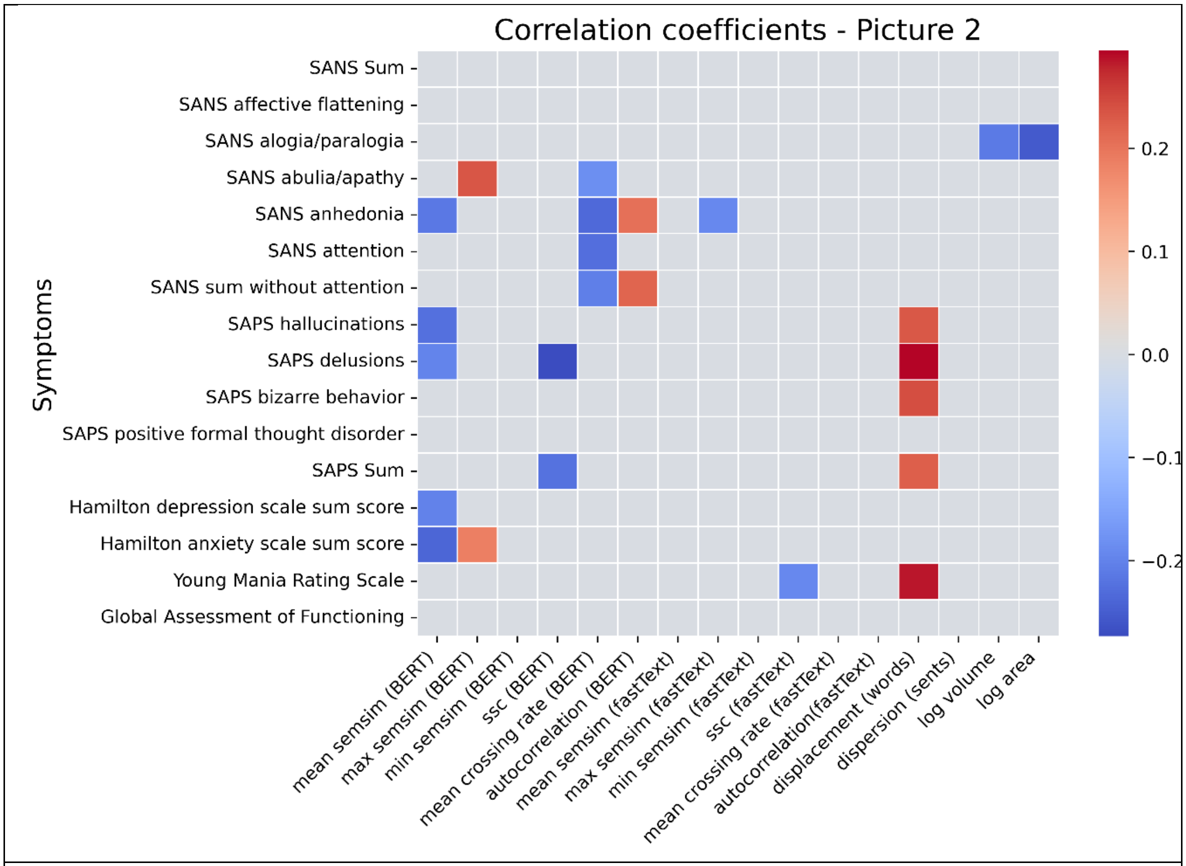

Correlation coefficients - Picture 2
Symptoms
SANS Sum
SANS affective flattening
SANS alogia/paralogia
SANS abulia/apathy
SANS anhedonia
SANS attention
SANS sum without attention
SAPS hallucinations
SAPS delusions
SAPS bizarre behavior
SAPS positive formal thought disorder
SAPS Sum
Hamilton depression scale sum score
Hamilton anxiety scale sum score
Young Mania Rating Scale
Global Assessment of Functioning
mean semsim (BERT)
max semsim (BERT)
min semsim (BERT)
ssc (BERT)
mean crossing rate (BERT)
autocorrelation (BERT)
mean semsim (fastText)
max semsim (fastText)
min semsim (fastText)
ssc (fastText)
mean crossing rate (fastText)
autocorrelation(fastText)
displacement (words)
dispersion (sents)
log volume
log area
0.2
0.1
0.0
−0.1
−0.2

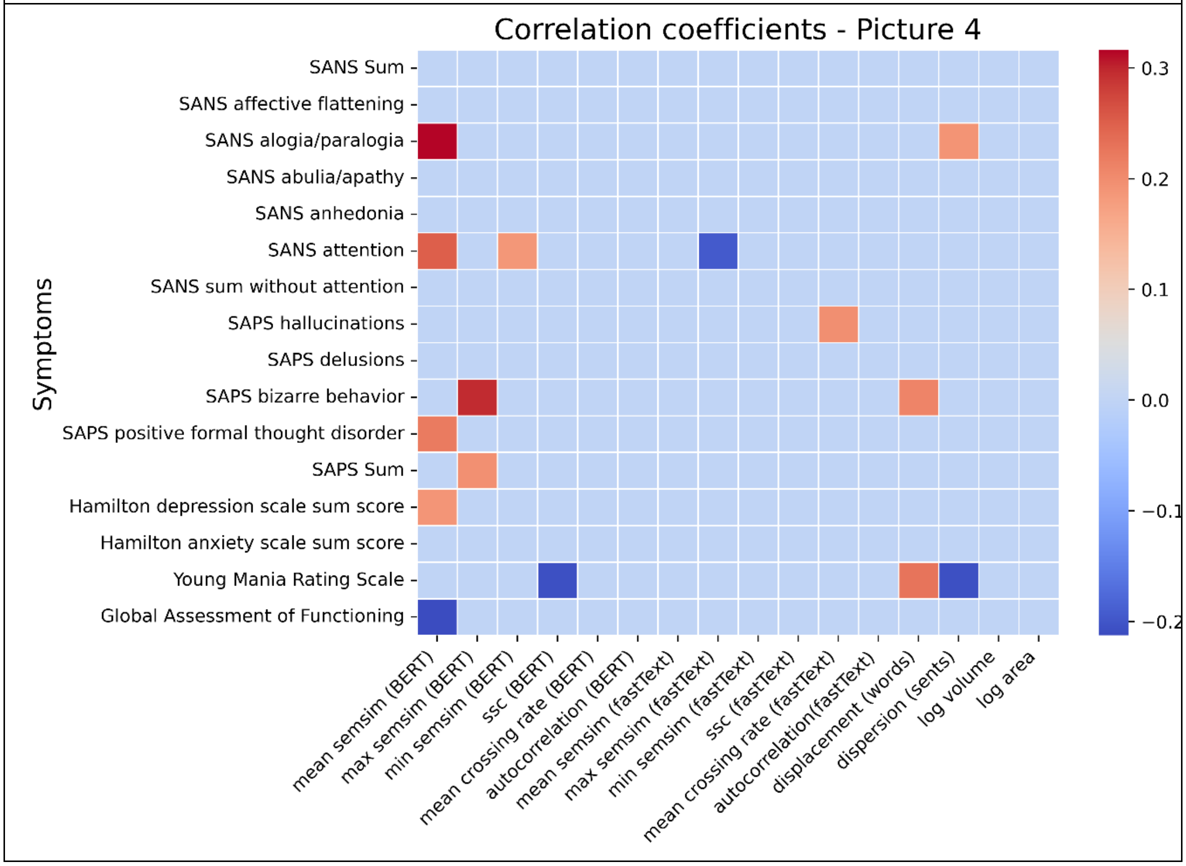

Correlation coefficients - Picture 4
Symptoms
SANS Sum
SANS affective flattening
SANS alogia/paralogia
SANS abulia/apathy
SANS anhedonia
SANS attention
SANS sum without attention
SAPS hallucinations
SAPS delusions
SAPS bizarre behavior
SAPS positive formal thought disorder
SAPS Sum
Hamilton depression scale sum score
Hamilton anxiety scale sum score
Young Mania Rating Scale
Global Assessment of Functioning
mean semsim (BERT)
max semsim (BERT)
min semsim (BERT)
ssc (BERT)
mean crossing rate (BERT)
autocorrelation (BERT)
mean semsim (fastText)
max semsim (fastText)
min semsim (fastText)
ssc (fastText)
mean crossing rate (fastText)
autocorrelation(fastText)
displacement (words)
dispersion (sents)
log volume
log area
0.3
0.2
0.1
0.0
−0.1
−0.2

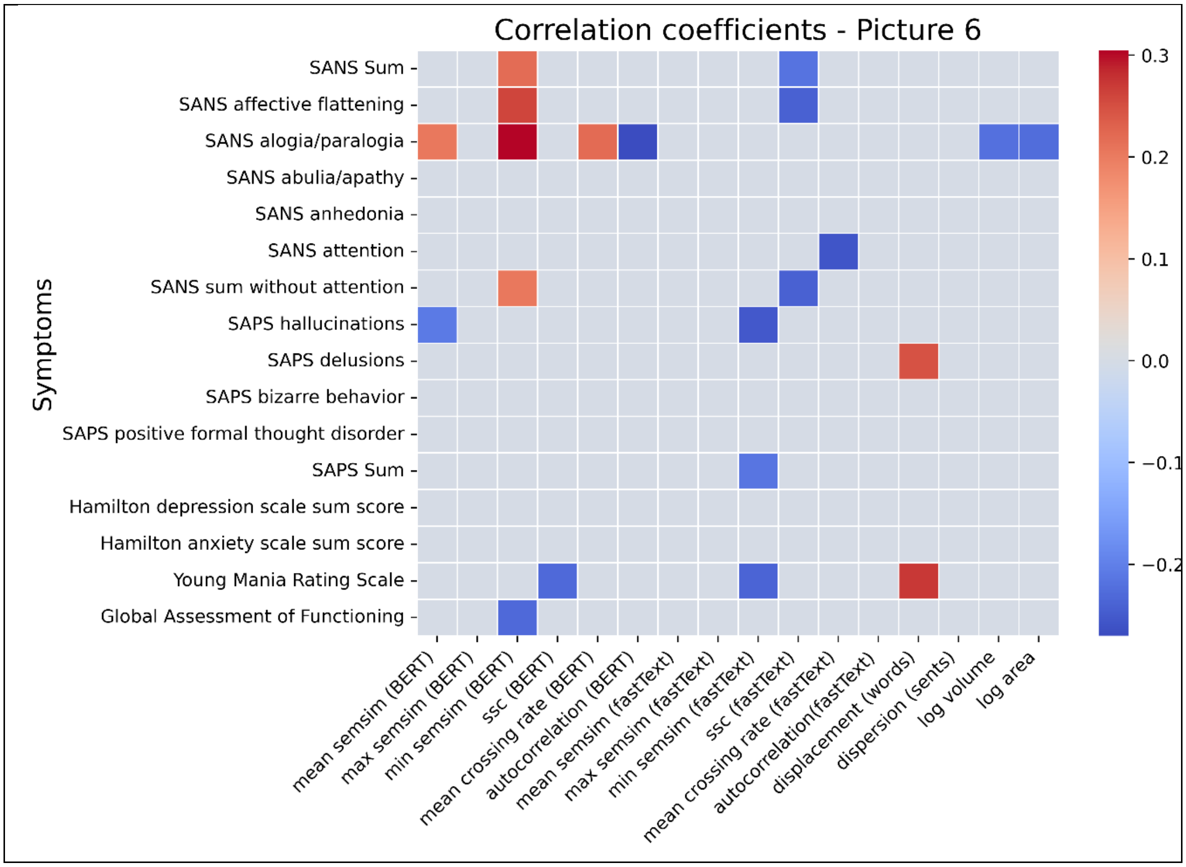

Correlation coefficients - Picture 6
Symptoms
SANS Sum
SANS affective flattening
SANS alogia/paralogia
SANS abulia/apathy
SANS anhedonia
SANS attention
SANS sum without attention
SAPS hallucinations
SAPS delusions
SAPS bizarre behavior
SAPS positive formal thought disorder
SAPS Sum
Hamilton depression scale sum score
Hamilton anxiety scale sum score
Young Mania Rating Scale
Global Assessment of Functioning
mean semsim (BERT)
max semsim (BERT)
min semsim (BERT)
ssc (BERT)
mean crossing rate (BERT)
autocorrelation (BERT)
mean semsim (fastText)
max semsim (fastText)
min semsim (fastText)
ssc (fastText)
mean crossing rate (fastText)
autocorrelation(fastText)
displacement (words)
dispersion (sents)
log volume
log area
0.3
0.2
0.1
0.0
−0.1
−0.2
